# Complete remission of classical Kaposi’s sarcoma following initiation of hormone therapy for prostate cancer

**DOI:** 10.1016/j.jdcr.2025.03.013

**Published:** 2025-03-29

**Authors:** Amélie Mourtada, Pierre Sohier, Nicolas Dupin

**Affiliations:** a Service de Dermatologie, Hôpital Cochin, ApHp, Université Paris Cité, Paris, France; b Department of Pathology, Hôpital Cochin, Assistance Publique-Hôpitaux de Paris, AP-HP.Centre-Université Paris Cité, Paris, France

**Keywords:** hormonal therapy, human herpes virus 8, Kaposi's sarcoma, prostate cancer

Kaposi's sarcoma (KS) is a rare opportunistic angioproliferative neoplasm caused by infection with human herpesvirus 8. Five epidemiological forms of KS have been characterized: classical KS, endemic KS, iatrogenic KS, AIDS-KS, and KS in men who have sex with men but are not HIV-infected.^1^ In some instances, KS is associated with immune dysfunction, as in AIDS KS or iatrogenic KS, where immune restoration alone can cure KS. In most cases, classic and endemic KS is an indolent disease, for which systemic treatment is unnecessary. However, for patients experiencing symptoms or aesthetic concerns, local treatments such as excision, radiotherapy, or chemotherapy injections may be required. Agressive forms, with visceral involvement or lymphedema, may develop. Chemotherapy is justified for the treatment of these forms, although the age of the patients, many of whom are old, may limit treatment options. Although the role of hormonal disparities has been evoked in KS pathogenesis as KS is predominantly observed in men, the effect of antiandrogenic treatment on KS has never been evaluated.

Our case report illustrated the benefit of antiandrogenic treatment for a prostate cancer on KS progression.

## Case report

**Fig 1:**
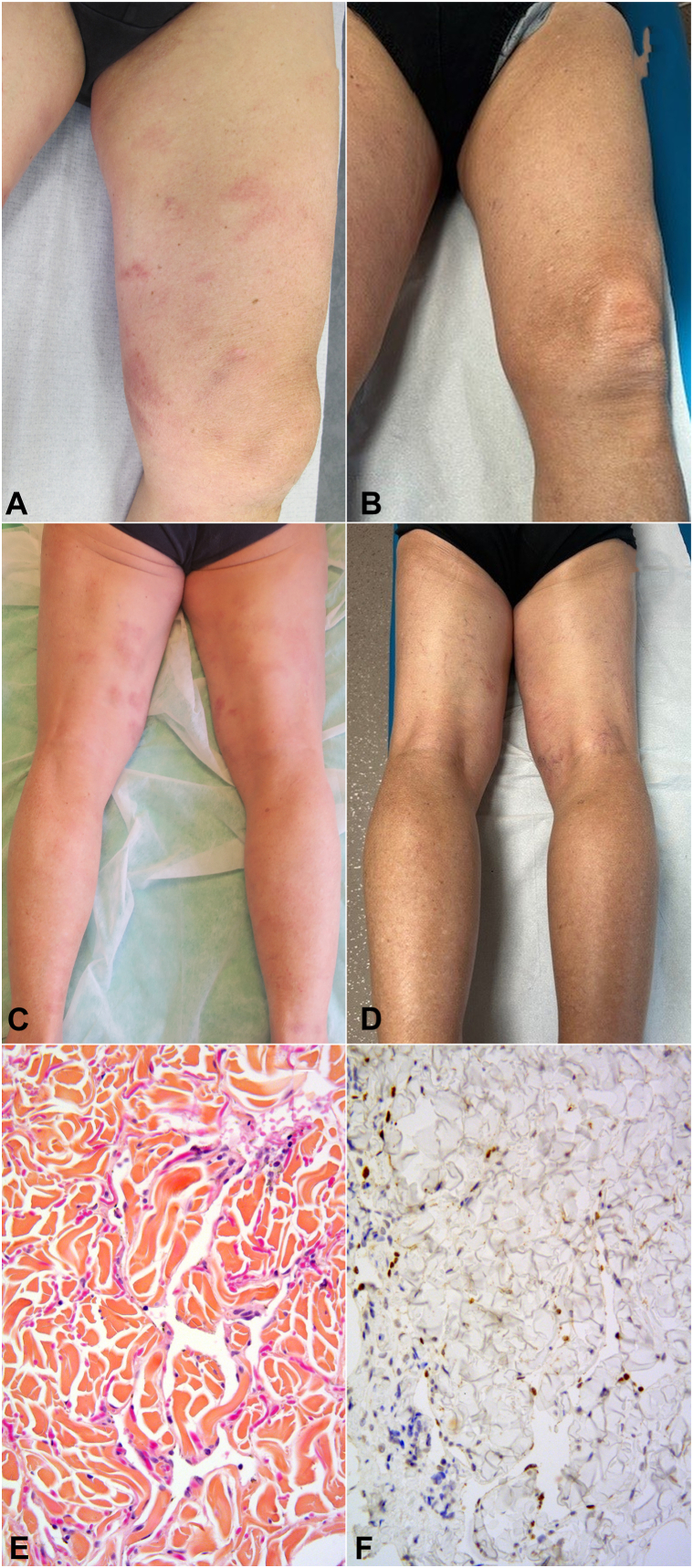
**A** and **C,** Kaposi sarcoma patch lesions of the limbs in a 81-year-old man before and complete remission after 2 years of initiation of an hormonal treatment for prostate cancer combining (**B** and **D**) oral abiraterone and injection of triptorelin. Subtle vascular proliferation dissecting the collagen bundles within the deep dermis showing anastomosing vascular channels lined by a single layer of endothelial cells. **E,** Hematoxylin and eosin saffron, 200× magnification. **F,** Immunohistochemistry with human herpesvirus type 8 (HHV-8) latent nuclear antigen-1 (LANA-1) antibody showing nuclear positivity on cells lining the vascular channels.

An 81-year-old man presented with a 13-year history of classical KS. He was of Armenian descent and had no history of HIV infection or immunosuppressive therapy. The initial KS lesions appeared at the age of 68, presenting as violaceous nodules on the feet. Five years later, the patient exhibited erythematous, finely scaly patches and plaques on the trunk and 4 limbs (Fig 1, *A* and *C*). Given this atypical clinical presentation, skin biopsies were performed on the abdomen, lumbar area, arm, and thigh. All skin biopsies showed a subtle dissecting vascular proliferation in the dermis, highlighted by nuclear positivity on immunohistochemistry with human herpesvirus 8 (HHV-8) antibody (Fig 1, *E* and *F*), confirming the diagnosis of KS. There was no visceral involvement on the computed tomography scan of the chest, the abdomen, and the pelvis. Given that the lesions were asymptomatic and stable, it was decided to continue therapeutic abstention with regular clinical monitoring.

After 11 years of follow-up, the patient was diagnosed with locally advanced prostatic adenocarcinoma and subsequently underwent focused external beam radiotherapy and antiandrogen hormone therapy. The latter consisted of a combination of subcutaneous triptorelin, a synthetic analog of the gonadotropin-releasing hormone, and abiraterone acetate, an androgen biosynthesis inhibitor associated with low-dose oral prednisone (5 mg daily). After 6 months of hormone treatment, the patient noted significant improvement of his skin lesions, and after 1 year, all lesions had disappeared. Complete clinical response was maintained at the last dermatology visit, after 18 months of treatment (Fig 1, *B* and *D*).

## Discussion

The strategy for treating KS depends on the possibility or nonpossibility of restoring immunity, which remains the best treatment. In classical KS, there is no possibility of modulating immunity, the disease is most often indolent, and due to the old age of the patients, it is better not to treat and to monitor the patient regularly. The decision for treatment can be taken in the event of functional discomfort or vital threat in the event of visceral damage, which remains rare. Classical KS predominantly affects men; however, the underlying mechanism for this gender disparity is poorly understood. Various observations have suggested the involvement of sex hormones in the development of KS lesions.^2^^,^^3^ *In vitro*, androgen receptors can upregulate the replication of HHV-8, both through an increased transcription of viral genes and by facilitating virus endocytosis.^4^^,^^5^ One can hypothesize that blocking androgen receptors can downregulate the replication of HHV-8, which is essential for infecting new endothelial cells and promoting KS pathogenesis. By inhibiting HHV-8 replication, antiandrogen therapy may lead to the regression of KS in this clinical context. In our observation, the link between the disappearance of KS and the initiation of hormonal treatment is highly probable given that the lesions had remained perfectly stable since the beginning of his follow-up.

To our knowledge, this is the first reported case of complete remission of KS under antiandrogen hormone therapy. This observation underlines a possible role of male sex steroids in KS. Targeting these hormones could represent a novel therapeutic approach in KS.

## Conflicts of interest

None disclosed.
